# The association between sarcopenia and frailty in older adults: a moderated mediation model

**DOI:** 10.1186/s12877-026-07529-0

**Published:** 2026-04-21

**Authors:** Shasha Gao, Huijun Zhang

**Affiliations:** Department of Nursing, Jinzhou Medical University, No.40, Section 3, Jinzhou City, Liaoning Province P. R. China

**Keywords:** Sarcopenia, Frailty, Functional limitation, Handgrip strength, Mediating effect

## Abstract

**Background:**

The prevalence of sarcopenia and frailty among older adults has emerged as a significant public health concern. Despite the recognition of their impact on health outcomes, the mechanisms linking sarcopenia to frailty remain inadequately explored. This study aimed to investigate the mediating role of functional limitations in the relationship between sarcopenia and frailty, while also examining the moderating effect of handgrip strength in this context.

**Methods:**

The sample for this study was derived from the 2015 China Health and Retirement Longitudinal Study (CHARLS), which offers nationally representative data. This dataset includes information from 21,097 individuals aged 45 and above, spanning around 150 regions and 450 villages across China. A sample of 2,885 individuals was extracted using RStudio. The outcome variable was frailty, the independent variable was sarcopenia, the mediator was functional limitation, and the moderator was handgrip strength. Spearman correlation analysis was conducted to examine the associations between the primary variables. The mediation and moderation effects were tested using PROCESS version 3.5.

**Results:**

In this study, the prevalence of sarcopenia was found to be 15.9% in the elderly population. Grip strength in older adults was negatively correlated with sarcopenia (ρ = − 0.241, *P* < 0.01), functional limitations (ρ = − 0.245, *P* < 0.01), and frailty (ρ = − 0.281, *P* < 0.01). Both sarcopenia (ρ = 0.156, *P* < 0.01) and functional limitations (ρ = 0.475, *P* < 0.01) showed positive correlations with frailty. Functional limitations were found to mediate the relationship between sarcopenia and frailty, accounting for 26.23% of the total effect. Additionally, grip strength moderated the initial pathway in the mediation model (β = -0.069, *P* < 0.05).

**Conclusions:**

The functional limitations in older adults mediated the effect of sarcopenia on frailty, and this mediating effect was moderated by handgrip strength.

**Supplementary Information:**

The online version contains supplementary material available at 10.1186/s12877-026-07529-0.

## Introduction

As global population aging accelerates, older adults increasingly confront various health challenges. Sarcopenia and frailty are prevalent conditions affecting this demographic, warranting significant scholarly focus [1–3]. Sarcopenia is defined as a substantial reduction in muscle mass and strength, attributed to factors such as aging, chronic disease, and lifestyle choices. This condition adversely impacts functionality and overall quality of life [4–6]. Conversely, frailty represents a multifaceted decline across several physiological systems, leading to decreased physical function and an impaired capacity to cope with stressors. Common manifestations of frailty include unintended weight loss, fatigue, and mobility limitations [7, 8]. Understanding the interplay between sarcopenia and frailty is essential for enhancing the quality of life among older adults, as their co-occurrence can exacerbate health complications, including falls, increased hospitalization rates, and elevated mortality risks [9].

Within this context, Activities of Daily Living (ADL) emerge as a crucial mediating factor. ADL encompasses the ability to perform fundamental daily tasks, such as bathing, dressing, and eating [10]. Research suggests that sarcopenia not only leads to a decline in muscle strength but also adversely affects the capacity for ADL, thereby heightening the risk of frailty [11–13]. Thus, functional impairment may mediate the relationship between sarcopenia and frailty, highlighting the specific ways in which sarcopenia contributes to physical decline in older individuals. This implies that improving ADL capabilities may serve as a strategic approach to mitigate frailty risk in elderly health management.

Additionally, handgrip strength plays a vital role as a significant moderating variable in this relationship. As an effective measure of muscle strength, handgrip strength also serves as a critical indicator of overall health in older adults. Studies have indicated that reductions in handgrip strength are closely linked to the progression of sarcopenia, directly influencing daily functioning and frailty status [14–16]. Handgrip strength not only reflects muscular health but also correlates with psychological well-being, self-efficacy, and social engagement in this population. Therefore, examining the moderating effect of handgrip strength on the relationship between sarcopenia and frailty can enhance our understanding of the complexities underlying functional status changes in older populations, offering new insights for health management and intervention strategies.

Although existing literature has established a robust correlation between sarcopenia and frailty, the underlying mechanisms-particularly the conditional pathways through which sarcopenia influences frailty-remain underexplored. Most previous studies have focused on direct associations or simple mediation models, without examining how individual characteristics, such as handgrip strength, may modify these relationships. To address this gap, the present study proposes a moderated mediation model that simultaneously tests (1) whether functional limitations mediate the sarcopenia-frailty relationship, and (2) whether handgrip strength moderates the pathway from sarcopenia to functional limitations. This approach moves beyond descriptive associations to elucidate the conditional mechanisms linking sarcopenia to frailty, thereby offering insights for personalized interventions.

Through this study, we aspire to clarify the intricacies of health deterioration in older adults and provide a theoretical foundation for formulating targeted intervention strategies aimed at improving the quality of life and health status within this population. The objective of this research is to systematically examine the interrelationships among sarcopenia, functional impairment, frailty, and handgrip strength, thereby offering scientific evidence and practical recommendations for effective health management in the elderly. For example, older adults with low handgrip strength may benefit most from resistance exercise programs targeting muscle power, whereas those with adequate strength might require different strategies such as balance or ADL training. This stratified approach moves beyond one-size-fits-all recommendations.

## Methods

### Study setting

This investigation utilized a cross-sectional framework, drawing on data from the 2015 China Health and Retirement Longitudinal Study (CHARLS). CHARLS serves as a nationally representative survey targeting individuals aged 45 and above in China. By 2024, five follow-up surveys had been conducted, encompassing sample populations from 150 counties and 450 communities or villages across 28 provinces, including autonomous regions and municipalities. A total of 12,241 households were chosen for the study, leading to successful interviews with 21,097 participants.

For this analysis, the 2015 wave was selected for two main reasons. Firstly, it contains all the core variables required for our moderated mediation model. These variables include sarcopenia (based on AWGS 2019 criteria), frailty (Fried phenotype), and functional limitations (ADL/IADL). The contemporaneous measurements in this wave ensure data consistency. Secondly, a substantial body of research on sarcopenia and frailty using CHARLS data has been based on the 2015 wave. This facilitates the comparability between our findings and the existing evidence base.

All procedures in this research adhered to the established CHARLS protocols and guidelines. Participants were enrolled in CHARLS voluntarily and provided informed consent before taking part. The original CHARLS project received ethical clearance from the Institutional Review Board at Peking University in June 2008 (IRB00001052–11015).

**Fig. 1:**
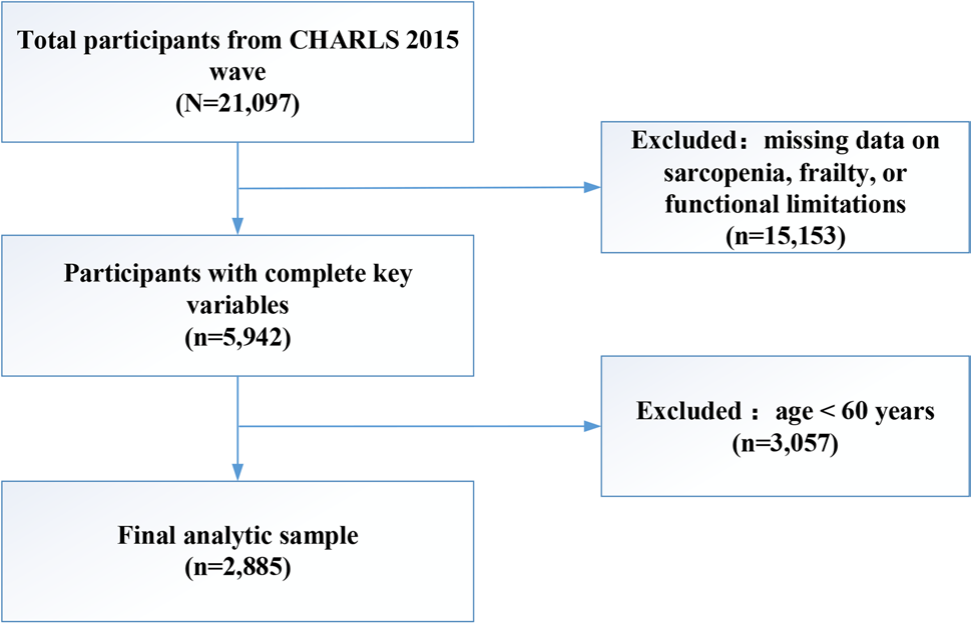
Flowchart of participant selection

From the 2015 CHARLS database, we initially included all participants who completed the main questionnaire and physical measurements (*N* = 21,097). We first excluded those with missing data on sarcopenia diagnosis, frailty, or functional limitations, leaving an intermediate sample of 5,944 participants. Subsequently, we excluded participants aged < 60 years, resulting in a final analytic sample of 2,885 participants. The detailed participant selection process is presented in Fig. 1.

## Measurements

### Sarcopenia

According to the AWGS 2019 criteria, sarcopenia is defined through the assessment of muscle strength, muscle mass, and physical function:

1. Muscle Strength: Measured using handgrip strength. A decline in muscle strength is indicated by handgrip strength values of less than 28 kg for men and less than 18 kg for women.
2. Muscle Mass: As CHARLS did not directly measure the muscle mass of respondents, this study estimated the appendicular skeletal muscle mass (ASM) based on the research conducted by Wen et al. The formula used is: ASM = 0.193 × weight (kg) + 0.107 × height (cm) − 4.157 × sex − 0.037 × age − 2.631. The appendicular skeletal muscle mass calculation employed a validated Chinese-specific algorithm, demonstrating strong concordance with DXA measurements [17, 18] .Additionally, muscle mass is evaluated using height-adjusted ASM (ASM/height²), with values of less than 7.0 kg/m² for men and less than 5.4 kg/m² for women indicating a decline in muscle mass.
3. Physical Function: Assessed using a 2.5-meter walk test and a five-time sit-to-stand test. A walking speed of less than 1 m/s or a completion time greater than 12 s in the five-time sit-to-stand test, or inability to complete the test, indicates a decline in physical function.

Sarcopenia is defined as a decline in muscle mass combined with either a decline in physical function or muscle strength. Severe sarcopenia is indicated by declines in muscle mass, physical function, and muscle strength. Individuals without any of these two conditions are classified as not having sarcopenia [19, 20].

### Frailty

The concept of frailty was first introduced by Fried et al. and encompasses several key indicators: unintentional weight loss, self-reported exhaustion, weakness, slow walking speed, and low physical activity levels. This study utilizes the frailty diagnostic criteria established in prior research [21].

The criteria under consideration include exhaustion, weakness, low physical activity, weight loss, and slowness. In this analysis, frailty is evaluated through the following specific measures:

1. Weakness was assessed via a self-reported question regarding difficulty lifting or carrying weights exceeding 5 kg [22].
2. Slowness was identified when a participant reported challenges in walking 100 m or climbing several flights of stairs without requiring a rest, a method consistent with previous studies [22].
3. Exhaustion was indicated if participants responded “Most or all of the time” or “Occasionally or a moderate amount of the time” to either of the relevant items from the Chinese version of the Center for Epidemiologic Studies-Depression scale (CES-D): “I felt everything I did was an effort during the last week” or “I could not get going during the last week.” This measure follows the original construction proposed by Fried et al. [23].
4. Low physical activity was determined if participants did not engage in physical activity or failed to walk for at least 10 min at a time during a typical week. Although this definition varies from that of Fried et al. [23], similar research has previously applied this measure to assess frailty [24, 25].
5. Weight loss was defined as an unintentional reduction of 5 kg or more within the past year or a current body mass index (BMI) of 18.5 kg/m² or lower. Evidence suggests that weight loss is a more reliable indicator of frailty than either BMI or energy intake.

In this study, we employed a modified version of the Fried frailty phenotype that assessed weakness via a self-reported question (“difficulty lifting or carrying weights exceeding 5 kg”) rather than objective grip strength measurement [26]. This modification was necessary to avoid conceptual overlap with handgrip strength, which was included as a continuous moderator in our model. Similar adaptations have been used in previous research using CHARLS data [27].

Frailty was ultimately defined by the presence of three or more of these five criteria [28].

### Functional limitation

We utilized the Activities of Daily Living (ADL) and Instrumental Activities of Daily Living (IADL) scales to assess functional limitations in older adults. The ADL scale assessed six basic tasks (dressing, bathing, eating, transferring, toileting, continence) based on the Katz Index of Independence in Activities of Daily Living [29]. The IADL scale assessed five instrumental tasks (housework, cooking, shopping, finances, medication) based on the Lawton Instrumental Activities of Daily Living Scale (Lawton IADL Scale) [30]. Both have been validated in Chinese older adults. Responses for both ADL and IADL were measured on a four-point scale ranging from 1 (no difficulty) to 4 (unable to perform). By summing the ADL and IADL scores, we formed a total score for functional limitations. Higher scores indicated a greater degree of functional limitation [31, 32].

### Handgrip strength

Handgrip strength was evaluated at baseline using a hydraulic handgrip dynamometer. Each participant’s left and right hands were measured two times. For the statistical analysis, we used the mean value from the four measurements as a continuous variable [33, 34].According to the Asian Working Group for Sarcopenia (AWGS) 2019 guidelines, the cut-off points for low handgrip strength were defined as < 28 kg for men and < 18 kg for women.

Although handgrip strength is a core component of sarcopenia diagnosis based on AWGS 2019 criteria, it was treated as a continuous moderator in our model to examine its dose-response effect on the sarcopenia-functional limitation pathway. This approach recognizes handgrip strength not only as a diagnostic threshold but also as an indicator of overall muscle function that may buffer the adverse effects of sarcopenia on daily activities.

### Control variables

Sociodemographic covariates included age (continuous), gender (male/female), marital status (single/married), education level (illiterate/primary school/junior high/high school and above), and place of residence (rural/urban).

Behavioral factors encompass smoking, drinking, and social activities, with each variable classified as “yes” or “no.”

Health status includes body mass index (BMI), the number of chronic conditions, and cognitive function. BMI is calculated from standard questions regarding height and weight. Chronic conditions are assessed based on self-reported diagnoses of 12 diseases, including hypertension, dyslipidemia, cancer, chronic lung disease, liver disease, myocardial infarction, stroke, kidney disease, asthma, mental health disorders, gastrointestinal diseases, and arthritis.

Cognitive function was assessed across several domains, including visuospatial abilities, memory, orientation, and attention. Visuospatial abilities were evaluated by having participants redraw an image consisting of two overlapping pentagons. A score of one point was awarded for correctly reproducing the image, while a score of zero was given for incorrect or incomplete drawings.

Memory performance was measured based on the average score from immediate and delayed recall of ten Chinese words. One point was assigned for each correctly recalled word.

Orientation and attention were assessed using the Telephone Interview of Cognitive Status (TICS-10). This tool assigns points based on responses to questions about the current year, month, day, day of the week, and season, as well as the serial subtraction of 7 from 100, repeated up to five times. One point was given for each correct response, with a total possible score ranging from 0 to 10.

The overall cognitive function score was derived by summing the individual scores across these dimensions, resulting in a total score ranging from 0 to 21, with higher scores indicating better cognitive performance [35].

Depressive symptoms were assessed using the 10-item Center for Epidemiologic Studies Depression Scale (CES-D). However, to avoid overlap with the frailty phenotype (which uses two CES-D items to define exhaustion), we calculated a modified CES-D score excluding the two exhaustion-related items. A score of 8 or higher on this modified 8-item scale (range 0–24) was considered indicative of depression [36]. Table 1 shows the definitions and codes of the variables.

**Table 1:**
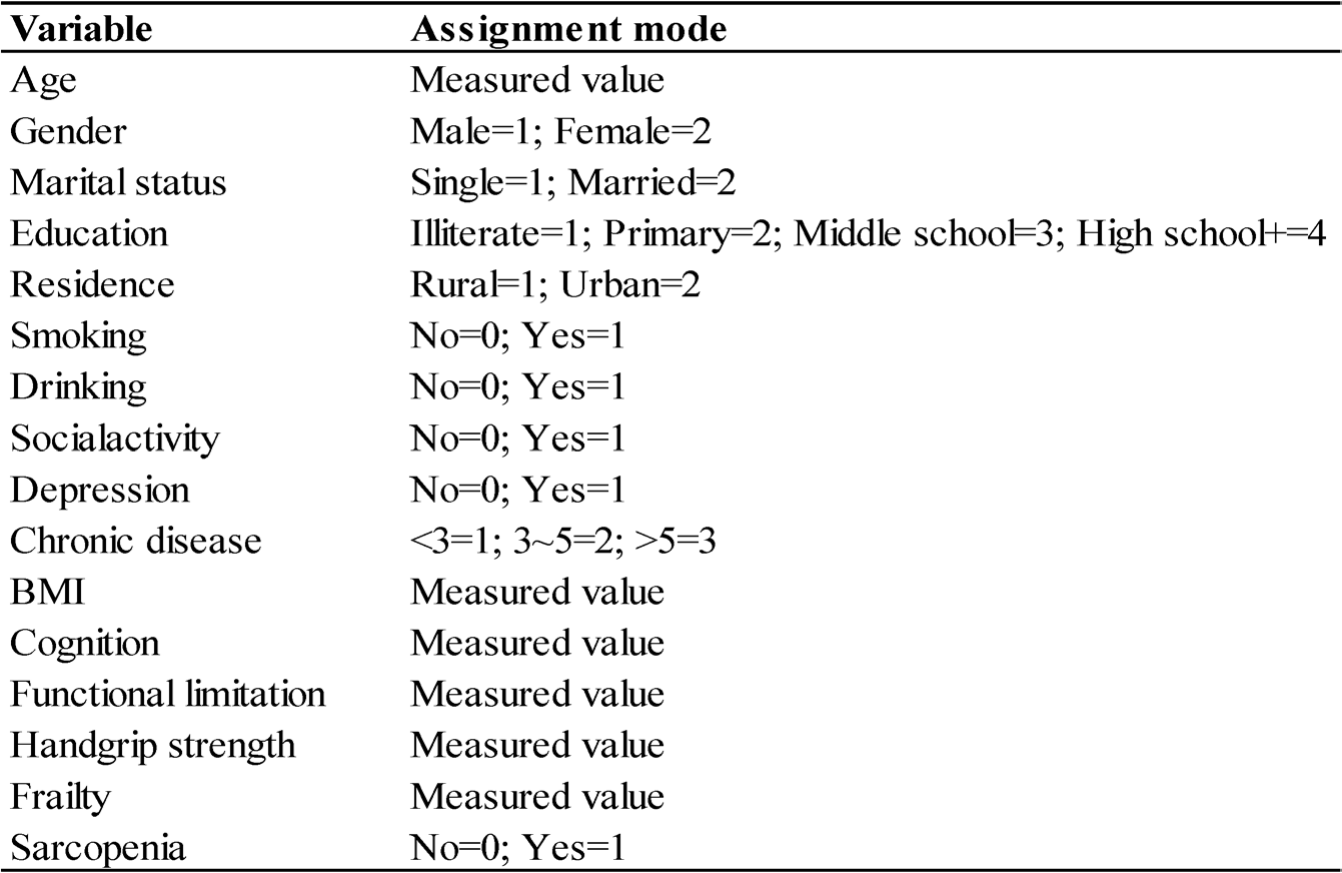
Case of variable assignment

### Statistical analysis

Data were processed using RStudio version 4.4.1 and SPSS version 25.0. Continuous variables were described using M (IQR)/Mean(SD), while categorical variables were presented as n (%). Spearman correlation analysis was employed to examine the relationships among sarcopenia, functional limitations, handgrip strength, and frailty. The mediating effect of functional limitations between sarcopenia and frailty in older adults was tested using Model 4 from PROCESS version 3.5, and Model 7 was utilized to assess the moderating effect of handgrip strength in the initial mediating pathway of sarcopenia on functional limitations. To assess potential multicollinearity, we calculated variance inflation factors (VIFs) for all independent variables; all VIF values were below 2, indicating no serious multicollinearity concerns. The significance level was set at α = 0.05.

## Results

### Sample characteristics

Among the 2,885 participants, the mean age was approximately 66 years, and the majority were female (81.8%). Most participants resided in rural areas, were married (78.2%), and had at least a high school education. Regarding health behaviors, most participants were non-smokers and non-drinkers, and the number of chronic diseases was predominantly fewer than three. Detailed information is provided in Table 2.

**Table 2:**
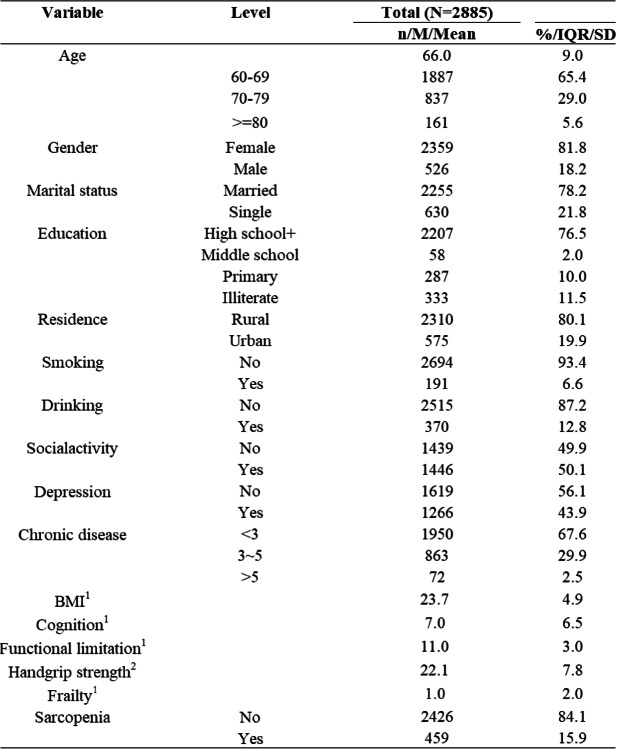
General characteristics of elderly people

Data are presented as *n* (%) unless otherwise indicated.^1^ Median (interquartile range).^2^ Mean ± standard deviation

### Correlation between key variables

The results of the Spearman correlation analysis indicate that grip strength in older adults is negatively correlated with sarcopenia, functional limitations, and frailty (*P* < 0.01). Additionally, both sarcopenia and functional limitations are positively correlated with frailty (*P* < 0.01), and there is a positive correlation between sarcopenia and functional limitations (*P* < 0.01), as detailed in Table 3.

**Table 3:**
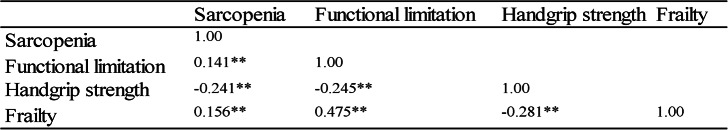
Correlations among sarcopenia, functional limitation, handgrip strength and frailty.

** P<0.01

### The mediating effect of functional limitations between sarcopenia and frailty in older adults

Frailty was treated as the dependent variable, sarcopenia as the independent variable, and functional limitations as the mediator. Age, gender, marital status, education level, living conditions, smoking, drinking, social activity, depression, BMI, cognitive function, and the number of chronic diseases were controlled for. Mediation analysis was conducted using Model 4 of the Process. The results indicated that sarcopenia had a significant positive predictive effect on frailty (*p* < 0.001). After including functional limitations as a mediator, the positive predictive effect of sarcopenia on frailty remained significant (*p* < 0.001), as shown in Table 4. The Bootstrapping results indicated that both the direct and indirect effects of sarcopenia on physical activity did not include zero in their 95% confidence intervals, confirming the existence of a mediation effect. The mediation effect was 0.085, accounting for 26.23% of the total effect, as shown in Table 5.

**Table 4:**
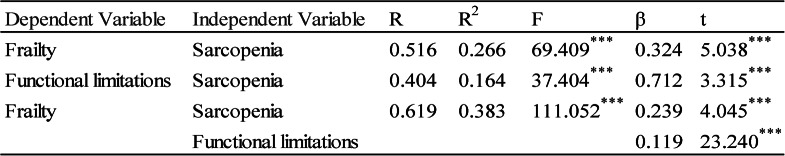
The mediating effect of functional limitations between sarcopenia and frailty in older adults

*** *P*<0.001

**Table 5:**
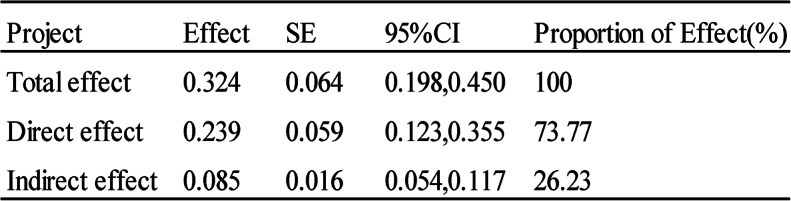
Testing the mediating effect of functional limitations between sarcopenia and frailty in older adults

### Handgrip strength as a moderator in the relationship between sarcopenia and frailty in older adults

**Fig. 2:**
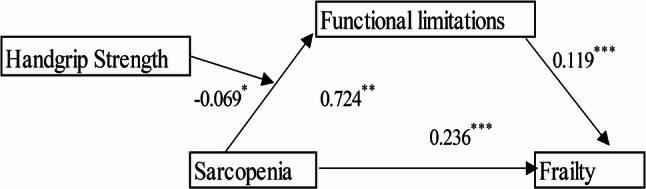
Moderation model of handgrip strength in the relationship between sarcopenia and frailty in older adults Notes: * *P* <0.05, ** *P* <0.01, *** *P* < 0.001

The moderation effect was tested using Model 7 in Process. The results indicate that after including handgrip strength in the model, sarcopenia positively predicted functional limitations (β = 0.724, *p* < 0.01); functional limitations positively predicted frailty (β = 0.119, *p* < 0.001); and sarcopenia positively predicted frailty (β = 0.236, *p* < 0.001). Furthermore, the interaction between sarcopenia and handgrip strength significantly predicted functional limitations (β = -0.069, *p* < 0.05). This suggests that the moderating effect of handgrip strength occurs in the initial pathway of sarcopenia predicting frailty. The moderation effect model is presented in Fig. 2. The results after applying the Bonferroni correction are presented in Supplementary Table S1.

**Fig. 3:**
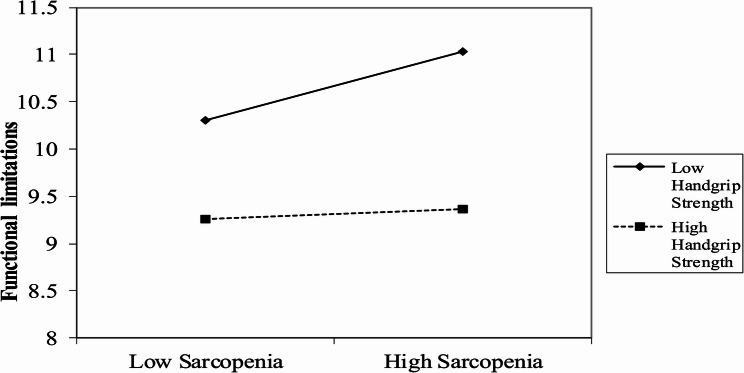
Simple slope plot

To examine the effects of the interaction terms in the moderation model, a simple slope analysis was conducted by categorizing handgrip strength into high and low levels. The high-level group was defined as one standard deviation above the mean, and the low-level group as one standard deviation below the mean, as shown in Fig. 3. The results revealed that at low handgrip strength levels, sarcopenia had a stronger impact on functional limitations, and this effect exerted a more substantial indirect effect on frailty through functional limitations. At high handgrip strength levels, the effect of sarcopenia on functional limitations was significantly weaker, and the indirect effect on frailty through functional limitations was no longer significant.

### Sensitivity analyses by gender

Because handgrip strength cutoffs differ between sexes, we re-ran the moderated mediation model separately for males (*n* = 526) and females (*n* = 2,359). The interaction term (sarcopenia × handgrip strength) was significant only in females (β = − 0.072, *P* < 0.05), possibly due to the smaller male sample size. When using gender-standardized handgrip strength, the results remained consistent. Percentages of low handgrip strength (AWGS cutoffs) were 84% female and 16% male and high handgrip strength group was 28% female and 72% male.

## Discussion

### The relationship between sarcopenia and frailty

The results of this study indicate that approximately 15.9% of the participants were diagnosed with sarcopenia. Previous studies have reported varying prevalence rates of sarcopenia in elderly populations. Our prevalence is higher than the 11.3% reported by Xu et al. [37] and 8.5% by Hu et al. [38]. This discrepancy may be attributed to differences in diagnostic criteria and population characteristics across studies. Existing research has shown that sarcopenia is a major risk factor for frailty, often leading to a decline in muscle strength, physical performance, and the ability to perform activities of daily living. Patients with sarcopenia typically exhibit limited physical mobility, insufficient endurance, and an increased risk of falls, all of which contribute to the acceleration of frailty development.

The association between sarcopenia and frailty has been confirmed in multiple studies. Previous research has indicated that sarcopenia, by reducing muscle mass and function, directly contributes to the onset of frailty, and is closely linked to adverse health outcomes in older adults, such as falls, fractures, and declines in activities of daily living. This study further confirms that sarcopenia is closely associated with frailty in this cross-sectional sample [39–41].

### The mediating role of functional limitation

This study also found that functional limitation plays a partial mediating role in the relationship between sarcopenia and frailty. The mediating path remained significant after Bonferroni correction, enhancing the credibility of the conclusion. Functional limitation is a critical indicator of an older adult’s ability to perform basic daily activities. In this study, the median functional limitation score was 11, with a quartile range of 3, and a score range from 11 to 39, suggesting significant variation in functional limitation among participants, with most individuals showing some degree of functional impairment.

Functional limitation is a key risk factor for frailty, and sarcopenia often impairs basic activities of daily living, thereby exacerbating functional limitations and accelerating frailty progression. Our findings confirm that functional limitation mediates this relationship, indicating that declines in muscle mass and function restrict daily activity capacity and subsequently intensify frailty.

Consistent with the findings of this study, existing literature has also indicated that declines in functional capacity are closely associated with frailty, particularly in those with sarcopenia, where functional limitation often signals the onset of worsening frailty [42–47]. These findings imply that comprehensive interventions should be implemented for older adults, especially those with sarcopenia. Interventions such as resistance training, increasing protein intake, and promoting greater daily physical activity can enhance muscle strength. Furthermore, functional training aimed at improving or restoring some level of daily functioning could effectively reduce the risk of frailty. A comprehensive approach not only helps improve overall health outcomes but also serves to delay the onset and progression of frailty.

### The moderating role of handgrip strength in the frailty process

Handgrip strength is an important indicator of overall muscle strength in older adults and is widely used in the assessment of frailty, sarcopenia, and related conditions. This study found that the median handgrip strength of the participants was 21.8 kg, with a quartile range of 9.4 kg, and a range from 0 to 76.5 kg, indicating significant variation in handgrip strength among the participants, with most individuals exhibiting low levels of handgrip strength.

The findings suggest that handgrip strength has a significant moderating effect on the relationship between sarcopenia and frailty. Specifically, while sarcopenia is common in older populations, stronger handgrip strength may help delay the onset of frailty and mitigate its severity. Individuals with greater handgrip strength typically exhibit better muscle function and higher levels of independence in daily activities, which can slow the progression of frailty. Consistent with these findings [48], previous research has highlighted that handgrip strength is closely associated with the overall health and quality of life in older adults, with individuals possessing stronger handgrip strength showing lower risks of frailty, falls, and dependency [14–16, 49, 50]. After Bonferroni correction, the weakening of the moderation effect may be due to the need for a larger sample size to detect the effect under the corrected threshold, or it may be due to the possibility of Type I errors in the uncorrected analysis.

Additionally, handgrip strength, as a reflection of overall muscle power, may contribute to improved endurance and physical capability, enhancing daily functional independence and further reducing frailty risk. Therefore, improving handgrip strength in individuals with sarcopenia not only enhances muscle mass but also serves as an important strategy for preventing frailty.

An important methodological consideration is the dual role of handgrip strength in this study: it is both a component of sarcopenia diagnosis and a moderator in our model. While this may initially appear circular, we argue that these roles are conceptually distinct. In sarcopenia diagnosis, handgrip strength is used as a binary threshold (below vs. above sex-specific cutoffs) to classify individuals. In contrast, as a moderator, handgrip strength was analyzed as a continuous variable to test whether its level modifies the strength of the association between sarcopenia and functional limitations. Our findings indicate that even among individuals with sarcopenia, higher handgrip strength attenuates the impact of muscle loss on daily function, highlighting its role as a protective reserve rather than merely a diagnostic criterion. This distinction is clinically meaningful: interventions targeting grip strength improvement may benefit older adults regardless of their sarcopenia status.

### Conceptual distinction of handgrip strength’s roles

An important methodological consideration is the dual role of handgrip strength in this study: in sarcopenia diagnosis it is used as a binary threshold, whereas as a moderator it is analyzed as a continuous variable. These roles are conceptually distinct. Our findings indicate that even among individuals with sarcopenia, higher handgrip strength attenuates the impact of muscle loss on daily function, highlighting its role as a protective reserve rather than merely a diagnostic criterion. This distinction is clinically meaningful: interventions targeting grip strength improvement may benefit older adults regardless of their sarcopenia status.

### Limitations

First, although we employed validated anthropometric equations for muscle mass estimation that demonstrate good agreement with dual-energy X-ray absorptiometry (DXA) and bioelectrical impedance analysis (BIA), inherent measurement limitations persist [51, 52]. Second, gait speed was assessed at 2.5 m rather than the conventional 6-meter distance. Existing evidence [53] suggests this abbreviated protocol yields comparable results in Chinese populations, as demonstrated by CHARLS 2015 validation studies. Third, our diagnostic approach lacked confirmatory clinical assessment for sarcopenia cases, potentially introducing classification bias. Fourth, although we adjusted for major known confounders, unmeasured factors including protein intake and dietary patterns were not accounted for due to data limitations in CHARLS. Fifth, the cross-sectional design prevents any causal or temporal interpretation. Future studies using two or more waves of CHARLS data are needed to examine longitudinal pathways. Additionally, recall bias inherent in self-reported questionnaires may influence our estimates. While the CHARLS 2015 data provided methodological advantages for this study, subsequent studies should include updated data (2018, 2020) to examine the temporal trends of these associations.

## Conclusions

Older adults with sarcopenia are often at a higher risk of frailty, and the presence of functional limitations worsens this risk. Handgrip strength, as an important physiological indicator, plays a moderating role in the relationship between sarcopenia and frailty. This study suggests that improving patients’ functional limitations can help alleviate the impact of sarcopenia on frailty, while enhancing handgrip strength can effectively moderate this relationship.

Therefore, it is recommended that healthcare professionals focus on assessing the degree of functional limitations and handgrip strength in older patients with sarcopenia and frailty. Focused rehabilitation and exercise programs designed to improve handgrip strength and functional ability can lower the risk of frailty and foster better overall health. This approach not only aids in improving the patients’ physical condition but also enhances their quality of life.

## Supplementary Information

Supplementary Material 1.

## Data Availability

All the data for this study can be accessed at the website.http://charls.pku.edu.cn/pages/data/2015-charls-wave4/en.html.
