# Supplementary material for: The association between sarcopenia and frailty in older adults: a moderated mediation model
Source: BMC Geriatr. 2026 Apr 21;26:792. doi: 10.1186/s12877-026-07529-0 (PMC13231568; doi:10.1186/s12877-026-07529-0)
Supplement: Supplementary file 1 — Supplementary Material 1. [file 12877_2026_7529_MOESM1_ESM.pdf]

Table S1 Results of Bonferroni Correction

Effect	β	Original p-value	Significance after Bonferroni correction
Sarcopenia [illegible] Functional limitatio	0.724	<0.01	Significant (p < 0.0125)
Functional limitations [illegible] Frailty	0.119	<0.001	Significant (p < 0.0125)
Sarcopenia [illegible] Frailty	0.236	<0.001	Significant (p < 0.0125)
Sarcopenia×Handgrip strength [illegible] Functional limitations	-0.069	<0.05	Non-significant (p > 0.0125)

Notes: Bonferroni correction formula($\alpha_{adj}=\alpha/n=$ 0.05/4=0.0125)
